# Exvivo Experiments of Human Ovarian Cancer Ascites-Derived Exosomes Presented by Dendritic Cells Derived from Umbilical Cord Blood for Immunotherapy Treatment

**DOI:** 10.4137/cmo.s776

**Published:** 2008-06-11

**Authors:** Qi-Ling Li, Ning Bu, Yue-Cheng Yu, Wei Hua, Xiao-Yan Xin

**Affiliations:** 1 Department of Gynecology and Obstetrics, Xijing Hospital, Fourth Military Medical University, Xi’an 710033, Shannxi Province, P.R. China; 2 Department of Neurology, Second Affiliated Hospital, Xi’an Jiaotong University, Xi’an 710004, Shannxi Province, P.R. China

**Keywords:** exosome, dendritic cells, umbilical cord blood, ovarian cancer, immunotherapy

## Abstract

**Objectives:**

Exosomes, a type of membrane vesicles, released from tumor cells have been shown to be capable of transferring tumor antigens to dendritic cells and activating specific cytotoxic T-lymphocytes. Recent work has demonstrated the presence of high numbers of exosomes in malignant effusions. Umbilical cord blood (UCB) is a rich source of hematopoietic stem cells and from which a significant number of dendritic cells can be produced. We hypothesized that the exosomes released from metastatic ovarian carcinoma were able to present tumor specific antigen to dendritic cells derived from unrelated umbilical cord blood, then could stimulate resting T cells to differentiate and induce effective cytotoxicity.

**Study design:**

Exosomes were isolated by ultracentrifugation of malignant ascites from ovarian cancer patients (n = 10). Purified exosomes were further characterized by Western blot analyses and immunoelectronic microscopy. Dendritic cells were collected from unrelated umbilical cord blood and cultured in the presence of GM-CSF, IL-4 and TNF-α. Resting T cells were mixed with dentritic cells previously primed with exosomes and the cytotoxicity were measured by MTT method. T cells were activated by DCs presented with exosomes.

**Results:**

1) the exosomes isolated from the ascites were membrane vesicles of about 30–90nm in diameter; 2) the exosomes expressed MHC class I molecules, HSP70, HSP90, Her2/Neu, and Mart1; and 3)umbilical cord blood-derived DCs previously exosome-primed stimulated resting T cells to differentiate and produce effective cytotoxicity.

**Conclusions:**

These results suggested that tumor-specific antigens present on exosomes can be presented by DCs derived from unrelated umbilical cord blood to induce tumor specific cytotoxicity and this may represent as a novel immunotherapy for ovarian cancer.

## Introduction

Exosomes are small membrane vesicles secreted into the extracellular compartment by exocytosis. Tumor cells secrete exosome-like vesicles. These subcellular membrane vesicles from endosomal origin are secreted upon fusion of multi-vesicular bodies with the plasma membrane [1, 2]. As a consequence, exosomes have a “cellular” membrane oriented with a limited variety of proteins derived from the cytosol, endocytic compartment membranes, and plasma membranes [3]. They are 30 to 90 nm in diameter, and may be involved in the communication between cells. The presence of proteins potentially involved in cell targeting and tumor antigen transportation in exosomes prompted us to hypothesize that tumor cell-derived exosomes could be antigen delivery systems allowing transfer tumor antigen from tumor cells to APC. Wolfers [4] demonstrated that tumor-derived exosomes in the supernatants of a tumor cell line contained and were capable of transferring tumor antigens to the DCs. Recent work suggested that tumor derived-exosomes are immunogenic [5] and exosomes may be a novel source of tumor-specific antigens which may be used for T-cell cross priming and be relevant for immuno-interventions.

Unrelated UCB offers many practical advantages as an alternative source of stem cells, including: 1) absence of risk for mothers and donors; 2) relative ease of procedure and greater availability comparing to unrelated bone marrow graft [6]; 3) the ability to store fully tested and HLA-typed cord blood in the frozen state, and availability for immediate use to transplant centers [7]; 4) a reduced likelihood of transmitting infections, particularly cytomegalovirus; 5) less stringent criteria for HLA matching for donor-recipient selection; 6) the absence of donor attrition and 7) a potentially reduced risk of GVHD [8].

Exosomes derived from tumor cell lines transfer shared tumor antigens to DCs and, thus stimulate T cells in an MHC class I dependent way, and provide cross-protection against syngeneic and allogeneic tumors in mice [9]. In this study, we hypothesized that the exosomes derived from ascites of the patients with ovarian cancer were able to present tumor specific antigen to unrelated umbilical cord blood-derived dendritic cells, to stimulate T cells to differentiate, and to induce effective cytotoxicity.

## Materials and Methods

### Patients

Ascites samples were collected from the inpatients in the OB and GY department of Xijing Hospital in Fourth Military Medical University. We included patients who presented with ovarian cancer associated with ascites and had tumor cells in the biological fluid. All patients have given their consents. Ascites were removed either at the first debulking operation or examination. We excluded patients who had received chemotherapy within 4 weeks before the removal of their ascites, had a concentration of protein in the exudates that was below 30g/L, or had haemorrhage associated with carcinomatosis.

### Preparation of umbilical cord blood samples

UCB samples were obtained from the umbilical cord of full-term babies from non-diabetic non-infectious disease mother according to conditions established by the OB&GY department of Xijing Hospital in Fourth Military Medical University. The cells were processed within 24 hours after collection.

### Isolation of exosomes

Ascites samples were centrifuged at 300 g to remove the floating cells and the supernatant were collected and subjected to subsequent centrifugation steps (at 800 g for 30 min, followed by 10,000 g for 30 min, and then 100,000 g for 1 h). The pellet was recovered and resuspended in a phosphate-buffered saline (PBS) solution and then subjected to differential centrifugation at 90,000 g for 1.25 h on a gradient column. The exosomes contained in the 30% sucrose/D_2_O cushion were recovered, resupended in phosphate-buffered saline, and concentrated by ultracentrifugation at 100, 000g for 1 h, as previously described [5]. The pellet was then resupended in saline and stored at −80 °C. The final quantification of exosomal proteins was measured by a CBA kit according to the manufacturer’s recommendations [10].

### Electron microscopy

Exosomes obtained after differential ultracentrifugation were fixed in 30μL Phosphorus tungstic acid (20g/L) and negatively stained for about 1 min. The stained exosomes were then examined under electronic microscopy. When indicated, we performed single immunogold labeling before the contrasting step using a monoclonal antibody to human MHC class I molecules (Soldano Ferrone, USA,-clone1A6);to Hsc70(Stressgen, Canada, -clone N27F3); to Hsp90 (Shanghai Universal Biotech Company, -clone 1A6); to MHC class molecules (Novocastra, UK, -clone 3B5); to Mart1(Novocastra, UK, -clone A103), or Her2/Neu (-clone ncb11). The antibodies were then visualized with protein A previously coupled to 5 nm gold particles (Boster, China).

### Western blot analysis

Exosomal or cell-lysate protein were extracted as described previously[4]. Exosomal or cell-lysate protein then were analyzed by western blotting using a monoclonal antibody to human. We analyzed them by Western blotting with a monoclonal antibody to human MHC class I molecules; to Hsc70; to Hsp90; to MHC class molecules; to Mart1/Melan A, or Her2/Neu. The signal was then detected using a horseradish peroxidase conjugated anti-mouse antibody and chemiluminescence detection kit.

### Preparing DCs from human umbilical cord blood

Fresh UCB samples were diluted 1:4 in PBS. After centrifugation in Ficoll-Hypaque (Tianjin Hematology Institute, China) at 400 r/min for 35 min, mononuclear cells were collected from the interface and washed twice in PBS by centrifugation at 200 r/min for 10 min. The mononuclear cells were then labeled with a CD34+ progenitor cell isolation kit (Miltenyi Biotec, Germany) and the CD34+ hematopoietic stem cells were obtained Mini magnetic cell sorting (Mini MACS, Miltenyi Biotec, Germany) according to the protocol. DCs were induced from cord blood CD34+ hematopoietic stem cells as previously described [11]. The differentiation of DCs was examined under a phase contrast microscope. On the 10th day, the tumor-derived exosomes were added to the DC culture and on the 14th day, the exosome-primed DCs were collected as effector cells (a).

### DCs surface phenotyping by flow cytometry

The following mAbs were used to characterize DCs surface phenotypes: PE-conjugated anti-CD11c (BD PharMingen, America); FITC-conjugated anti-CD80 (BD PharMingen, America); PE-conjugated anti-CD86 (BD PharMingen, America), FITC-conjugated anti-HLA-DR(BD PharMingen, America). The harvested exosome-primed DCs were washed, resuspended in PBS supplemented with 1% BSA and 0.01% NaN_3_, and incubated with the mAbs for 30 minutes on ice. The cells were washed twice in PBS and 1 × 10^4^ of those labeled cells were subjected to FACS flow cytometric analysis using a Cellquest software.

### Isolation of epithelial ovarian cancer cells and T cells from ascites

Ovarian cancer cells were isolated from freshly collected malignant ascites as described previously [12]. T cells were isolated from the ascites as previously described [13]. T cells (at a density of 2 × 10^5^ cells/well) were cultured with effector cells (a) (at a density of 1 × 10^4^ cells/well) in 96-well culture plate for 48 h, as effector cells(b).

### Assessment of cytotoxicity by MTT assay

The ovarian cancer cells were seeded at a concentration of 5 × 10^4^ cells/ml in a 96-well culture plate. Autologous effector cells(b) were added according to variable concentration. Ovarian cancer cells from ascites were used as blank control cells. After 48 hrs of culture, 3-[4,5-dimethylthia zol-2-yl]-2,5- diphenyltetrazolium bromide (MTT) was added at a concentration of 0.5 mg/ml and incubated at 37 °C in CO_2_ incubator for an additional 24 hrs. Viable DCs generated insoluble crystal, but dead DCs were floating or loosely attached on the surface of culture plates. 10% SDS solution containing 0.01 N HCl was directly added into wells (100 L/well) to dissolve the insoluble crystal generated by DCs and to avoid the potential loss of samples. After 24 hrs, the absorbance of each sample was measured at 490 nm, using a microplate reader and the absorbance of each sample, 570 nm was used as reference.

### Statistical analysis

Data were analyzed with SPSS10.0 statistical software. Two-sided P < 0.05 was considered statistically significant.

## Results

### Sucrose gradient ultracentrifugation

**Figure 1:**
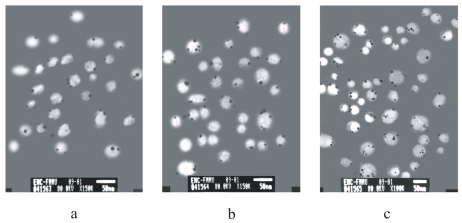
Immunoelectron micrograph of exosomes labeled with antibodies to (**a**) MHC class I molecules, (**b**) Her2/Neu, and (**c**) Mart1. Black dot indicated the presence of the 5 nm gold particles.

After overnight centrifugation of the fluorescent-labeled ascites, various yellow-white layers appeared in the sucrose gradient. In all samples, electronic microscopic analysis of these fluorescent fractions confirmed the presence of round homogeneous membrane vesicles which fulfilled the definition of exosome. Some variations in both shape and diameter of exosomes were observed among samples. The concentration of exosomes in fluorescent fractions varied even between samples from the same patient and the exosomes were occasionally mixed with other types of cell membrane fragments. When antibodies of MHC class I molecules, Her2/Neu and Mart1 were used to label the exosomes, gold particles were seen by immunity electronic microscopy (Fig. 1), whereas the sample labeled with antibodies to HSP70 or Hsp90 did not show any gold particles.

### Western blot analysis

**Figure 2:**
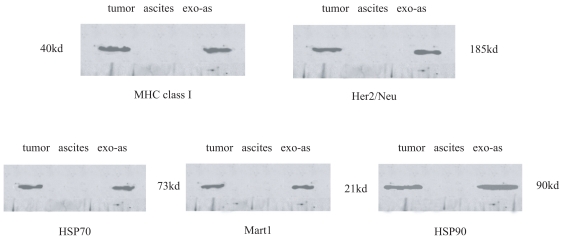
The presence of MHC class I molecules, HSP70, HSP90, Her2/Neu and Mart1 was confirmed by Western blotting in the exosome containing fraction isolated from malignant pleural fluid from patients with ovarian cancer. (tumor: lysated cells of ovarian cancer; ascites: ascites from the patients with ovarian cancer; exo-as: exosome derived from ascites of the patients with ovarian cancer).

Western blot analysis of the exosomes isolated from patients (n = 10) showed the presence of MHC class I molecules, HSP70, Hsp90, Her2/Neu, and Mart1(Fig. 2).

### Generation of DCs from cord blood CD34**+** cells

CD34+ isolated cells from cord blood were round and regular with a diameter of approximately 7–8 μm. Upon stimulation with rhGM-CSF, IL-4, and rhTNF-α, the cell number increased and cell clone formed. The cells also produced cytoplasmic projections. During the late period of culture, DCs shed from the clones into the medium. On day 14, the total number of cells increased by about 20-fold.

### DCs surface phenotyping by flow cytometry

**Figure 3:**
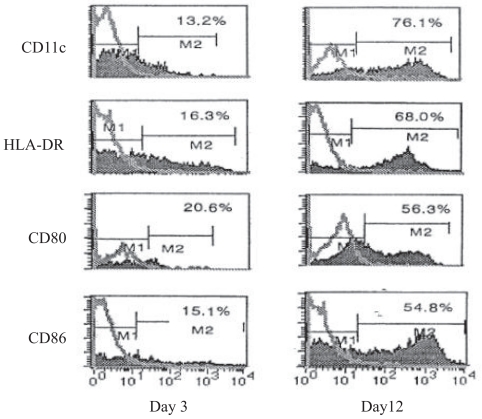
Flow cytometric analysis of phenotypic changes in DCs upon *in-vitro* stimulations with rhGM-CSF, IL-4 and TNF-α on day 3 and day 12.

The expression of CD11c, HLA-DR, CD80 and CD86 was gradually increased in DCs stimulated with rhGM-CAF, rhIL-4, and TNF-α. On day 3, approximately 13.2%, 16.3%, 20.6%, and 15.1% of DCs expressed CD11c, HLA-DR, CD80, and CD86, respectively and these numbers increased to 76.1%, 68.0%, 56.3% and 54.8% on day 12 (Fig. 3).

### Cytotoxicity of DC-primed lymphocytes in vitro

Lymphocytes were primed by exosomes pulsed-DCs at responder-to-stimulator ratio of 10:1, 20:1and 30:1 respectively. As shown in Table 1 and Table 2, the neutral red absorbance value(A value) of viable tumor cells at ratio of 30:1 in exo-DC-T group and exo-T group was significantly lower than that in DC-T and T groups (P < 0.01). There was no significant difference in A value between DC-T group and T group (P > 0.05). According to A value, the cytotoxicity of exo-T group or exo-DC-T group was 40.09% or 53.92% respectively, while the cytotoxicity without exosomes was 13.87% and 19.75%.

## Dicussion

Ideally, immunotherapeutic strategies aimed at immunizing the host should be able to elicit T-cell–mediated immune responses against a broad repertoire of tumor specific antigens. While mature DCs appear to be the most potent natural adjuvants, optimal protocols leading to efficient DC uptaking, processing, and cross-presentation in association with MHC class I molecules, are still lack. Several approaches involving the use of whole tumor RNA, tumor lysates, apoptotic or necrotic debris and fusion are currently under investigation [4]. Exosomes are released in vitro by many types of cells including tumor cell lines [4] and antigen presenting cells [1, 14]. These membrane vesicles have a different spectrum of proteins compared with plasma membranes and are enriched in molecules involved in antigen presentation and proteins involved potentially in cell targeting. Recent work suggested that tumor-derived exosomes contain a spectrum of tumor specific antigens and play a role in immunotherapy directed towards malignant tumors [5]. When exosomes are combined with source DCs, the exosomes probably interact directly with cognate receptors on the T cells via costimulatory molecules, and therefore induce more efficient T-cell activation [15]. Plasmacytoma cells were shown to release exosomes in vitro, and vaccination with a single dose (5 μg) of exosomal protein protected 80% of mice against challenge with wild-type tumors. The protection was likely to be related to the immune system since vaccinated mice generated specific cytotoxic T lymphocytes, the effects were not seen in SCID mice, and immunity was tumor-specific [16].

Exosomes present in malignant ascites may have various cellular origins. Malignant ascites is accompanied by a strong inflammatory response involving both cellular and humoral immunity. The most abundant cells in the ascites of our 10 patients were lymphocytes and tumor cells. B-lymphocytes, T-lymphocytes, and antigen-presenting cells such as dendritic cells are important participants of the anti-tumoral immune response, and are all known to produce exosomes [1, 17]. Moreover, tumor cells are also able to produce exosomes [3, 4, 18, 19].

Ovarian cancer cells are also able to produce exosomes [20, 21]. In this study, the exosomes isolated from body fluids expressed MHC class I molecules, HSP70, HSP90, Her2/Neu and Mart1, similar to tumor cells, when examined with western blotting and electronic microscopy. Our results suggest that carcinomatous ascites contain a combination of exosomes of ovarian cancer-origins. We further determined the MHC class concentration on the exosomes. Our results demonstrated that the exosomes isolated from the carcinomatous ascites contained MHC molecules (class I but not class II) and heat short proteins. Our results were in line with previous reports that these molecules were commonly identified in exosomes originating from various cells such as B cells, T cells, dendritic cells, and tumor cells [4,14,22] and recent work by André and colleagues showing the presence of MHC class I molecules in malignant ascites-derived exosomes by electron microscopic immunostaining and Western blotting [18]. Functions of these proteins in exosomes have been related to their capacity to transfer antigens to antigen presenting cells and to induce a specific immune response. These results prompted us to hypothesize that exosomes derived from ascites could be administered as a novel source of specific antigens in the development of immunotherapy.

To test our hypothesis that exosomes derived from ascites could be administered as a novel source of specific antigens in the development of immunotherapy, we performed the following experiments to determine if the exosomes with tumor antigens and costimulatory molecules isolated from ascites of patients with ovarian cancer could directly activate neonatal T cells and produce cytotoxicity. Our results showed the cytotoxicity of Exo-DC-T group was higher than that of Exo-T group and the DCs isolated from unrelated UCB were more efficient of activating T cells and producing more cytotoxicity when these DCs were primed with malignant ascites-derived exosomes. In conclusion, cancerous ascites contains exosomes from tumor cell origin. The proteomic analysis of ascites-derived exosomes in this study demonstrated that these exosomes expressing a variety of proteins including MHC class I molecules, HSP70, Hsp90, Her2/Neu, and Mart1 which are potentially involved in antigen presentation and activation of T-cell dependent immunity. Our current study also demonstrated that large number of DCs could be isolated from unrelated UCB and used as APCs to activate resting T cells and lead to effective cytotoxicity in the presence of malignant ascites-derived exosomes.

## Figures and Tables

**Table 1:** The efficiency of exosome purification process for 10 samples of ascites.

	Ascites parameters and final yield of exosomes as related to total number of MHC class I molecules									
	Sample 1	Sample 2	Sample 3	Sample 4	Sample 5	Sample 6	Sample 7	Sample 8	Sample 9	Sample 10
Starting volume(ml)	1240	930	1500	3000	1500	1700	1600	2000	1500	1500
Final volume(ml)	5.8	5.0	7.5	1.5	7.5	8.0	8.0	9.5	7.5	7.0
Fold concentration	214×	186×	200×	200×	200×	213×	200×	210×	200×	214×
Total exosomal MHC class I mole purified (×10^−14^)	2.4	1.6	3.2	1.2	3.7	2.4	3.2	2.5	3.1	1.5
Exosomal MHC class I/mg protein (×10^−14^) purified	1.27	0.34	0.96	0.21	0.73	1.34	1.20	0.97	0.96	0.86

Total protein(mg) = (volume after processing) × protein concentration determined by BCA assay.

**Table 2:** Neutral red absorbance value (A value) of ovarian cancer cells from ascites and cytotoxicity of effector cells in different groups at responder-to-stimulator ratio of 30:1(mean ± SD).

Group	A	Cytotoxicity (%)
Control	0.1369 ± 0.0145	
T	0.1087 ± 0.0256^a^	13.87
DC-T	0.0945 ± 0.0345^a^	19.75
exo-T	0.0528 ± 0.0145^b^,^c^	40.09
exo-DC-T	0.0370 ± 0.0136^b^,^d^,^e^	53.92

a P < 0.05 vs control group,

b P < 0.01 vs control group,

c P < 0.01 vs T group,

d P < 0.01 vs DC-T group

e P < 0.05 vs exo-T group.
